# Assessment of Antioxidant Potential of Carbon-Based Nanomaterials from Different Sources

**DOI:** 10.3390/antiox14101227

**Published:** 2025-10-13

**Authors:** Oladoyin Grace Famutimi, Sam Masha, Rodney Maluleke, Vuyelwa Ncapayi, Thabang Calvin Lebepe, Nande Mgedle, Cynthia Mutendu Kungwa, Olufunto Tolulope Fanoro, Isaac Olusanjo Adewale, Oluwatobi Samuel Oluwafemi

**Affiliations:** 1 Department of Biochemistry and Molecular Biology, Obafemi Awolowo University, Ile-Ife P.O. Box 17011, Nigeria; ofamutimi@oauife.edu.ng; 2 Department of Chemical Sciences (Formerly Applied Chemistry), University of Johannesburg, Doornfontein, Johannesburg 2028, South Africa; 201494994@student.uj.ac.za (S.M.); rmaluleke@uj.ac.za (R.M.); 216087577@student.uj.ac.za (V.N.); tlebepe@uj.ac.za (T.C.L.); 220165136@student.uj.ac.za (N.M.); 218094905@student.uj.ac.za (C.M.K.); 217072042@student.uj.ac.za (O.T.F.); 3 Centre for Nanomaterials Sciences Research, University of Johannesburg, Doornfontein, Johannesburg 2028, South Africa

**Keywords:** antioxidant, carbon dots, oxidative stress, nanomaterials

## Abstract

Antioxidants regulate oxidative reactions by impeding, delaying, or inhibiting the oxidation of biomolecules. Concerns regarding the toxicity of synthetic antioxidants have driven the search for safer alternatives. In this study, the antioxidant activities of three nontoxic carbon-based nanomaterials—carbon dots from citric acid precursor (CB-Ca), iron-doped carbon dots (CB-Fe) and carbon dots derived from *Momordica charantia* leaves (CB-Mc)—were investigated using 2,2-diphenyl-1-picrylhydrazyl (DPPH) radical scavenging, hydrogen peroxide (H_2_O_2_) scavenging, ferric-reducing antioxidant power, and total antioxidant capacity (TAC) assays. Scavenging activity was carried out at varying concentrations, and half-maximal inhibitory concentration (IC_50_) was calculated using non-linear regression. Reductive ability and TAC were expressed as mg ascorbic acid equivalents/g nanomaterial. CB-Fe exhibited the most potent DPPH scavenging activity (IC_50_ = 254.2 ± 37.37 µg/mL), surpassing CB-Mc and CB-Ca by 2- to 3-fold. In contrast, CB-Ca had the highest H_2_O_2_ scavenging (IC_50_ = 84.2 ± 11.87 µg/mL), while CB-Mc had the highest TAC of 77.95 mg ascorbic acid Eq/g. CB-Fe also displayed superior ferric ion reducing capacity. The study concluded that each carbon dot type exhibits unique antioxidant profiles and may offer some special advantages in nanomedicine and other applications.

## 1. Introduction

Reactive oxygen species (ROS) and reactive nitrogen species (RNS) are chemically reactive molecules derived from oxygen and nitrogen. They include both free radicals, which contain unpaired electrons, and non-radical reactive derivatives. These species are generated endogenously during normal cellular processes such as mitochondrial respiration and enzymatic reactions involving NADPH oxidases, nitric oxide synthase, and xanthine oxidase, as well as through immune responses [1]. They can also arise from exogenous factors such as pollutants, xenobiotics, and radiation exposure. Their high reactivity enables them to modify biomolecules, including lipids, proteins, and nucleic acids, leading to structural alterations and functional impairment [2].

At physiological levels, ROS and RNS play critical roles in maintaining homeostasis by mediating cellular signalling, host defence mechanisms, and vascular regulation. However, when their production exceeds the neutralising capacity of antioxidant defences, oxidative and nitrosative stress occur. This imbalance is toxic to cells and has been implicated in the pathogenesis of several diseases, including diabetes, Alzheimer’s disease, rheumatoid arthritis, and cancer [3].

Reactive oxygen and nitrogen species are byproducts resulting from cellular redox processes and encompass free radicals like hydroxyl (OH), superoxide (O_2_^•−^), alkoxy (RO), peroxyl (ROO), nitroxyl anion (NO^−^), nitrosonium cation (NO^+^), S-nitrosothiols, and dinitrosyl iron complexes. Under normal conditions, they play essential roles in various physiological processes and cellular signalling. However, their excessive levels can lead to significant oxidative stress-related damage, contributing to a range of diseases such as diabetes, Alzheimer’s disease, rheumatoid arthritis, and cancer [4]. Therefore, regulating reactive oxygen and nitrogen species concentrations in living organisms and maintaining them within a proper range is crucial to normal body homeostasis and mitigating diseases associated with oxidative irregularities [5]. Antioxidants, also known as free radical scavengers, help alleviate oxidative stress induced by free radicals by providing additional electrons [6].

Antioxidants also play a vital role in regulating oxidative reactions by impeding, delaying, or inhibiting the oxidation of biomolecules [7]. Specific components within key antioxidant enzymes act as protective shields for proteins. Furthermore, non-enzymatic antioxidants, such as water-soluble vitamins like vitamin C and glutathione, as well as fat-soluble vitamins such as vitamin E and beta-carotene, as well as synthetic antioxidants like butylated hydroxytoluene (BHT) and butylated hydroxyanisole (BHA), possess the capability to neutralise free radicals [8]. However, there are concerns of recent regarding the synthetic ones as being harmful to humans, necessitating the quest for more efficient, nontoxic compounds such as quantum dots with antioxidant properties [9]. Nanomaterials have been explored as replacements due to their significant benefits in a variety of biomedical applications, such as drug delivery, medical imaging, disease diagnosis, cancer treatment, infectious disease management, and treatments for neurodegenerative disorders like Parkinson’s disease [10]. Antioxidant therapies with nanomaterial integration hold the promise of a more focused and all-encompassing therapeutic approach. Carbon-based nanomaterials have emerged as a highly promising and adaptable class of materials with a broad range of applications spanning multiple scientific and technological domains in recent times [11]. These nanomaterials, which are primarily made of carbon atoms, have remarkable qualities and characteristics that make them excellent choices for solving problems such as cell and tissue imaging in theranostics; environmental remediation for the removal of organic molecules, oil, and heavy metals in water bodies [12].

Some of their characteristics include remarkable mechanical strength, exceptional electrical conductivity, high thermal stability, and intriguing optical properties. They differ from bulk materials in that they can display quantum effects due to their distinct structural arrangements at the nanoscale. Their biocompatibility and adjustable surface chemistry make them very appealing for applications in the fields of materials science, biomedicine, and environmental remediation [13]. Omran and Baek [14] highlighted how nanoantioxidants, including carbon nanomaterials, exhibit radical-scavenging activity strongly influenced by physicochemical factors such as particle size, charge, crystallinity, and surface functionalisation, and further emphasised the advantages of bio-based synthesis routes for improving stability and biocompatibility. Tang et al. [15] reviewed advances in low-dimensional carbon materials, including fullerenes and graphene quantum dots, and showed that structural features such as cage size, heteroatom doping, and functional group modification enhance their ability to neutralise reactive oxygen species. For example, carbon dots prepared from garlic and *Gynostemma* efficiently eliminated excess ROS in cells and zebrafish, while glutathione–citric acid-derived carbon dots scavenged DPPH, hydroxyl, and superoxide radicals.

To comprehensively assess antioxidant potential, no single assay is sufficient, as different mechanisms of free radical neutralisation may dominate depending on the extract or nanomaterial studied. Therefore, we employed four complementary assays, including: DPPH radical scavenging, hydrogen peroxide (H_2_O_2_) scavenging, ferric-reducing antioxidant power (FRAP), and total antioxidant capacity (TAC). These methods were chosen on the basis of their distinct antioxidant mechanisms of action: DPPH evaluates the capacity to donate electrons or hydrogen atoms to stabilise free radicals; H_2_O_2_ scavenging reflects the ability to directly eliminate a physiologically relevant reactive oxygen species; FRAP quantifies reducing power by electron transfer to ferric ions; and TAC provides an integrated estimate of overall antioxidant capacity, encompassing both hydrophilic and lipophilic contributors [16,17]. Together, these assays will provide a more holistic and informative evaluation of antioxidant activity.

We herein report the antioxidant properties of carbon dots and Fe-doped carbon dots derived from citric acid precursor and carbon dots derived from *Momordica charantia* leaves, and their effectiveness in mitigating oxidative stress by scavenging free radicals. Though there have been some reports on the antioxidant properties of some carbon-based materials [14,15,18,19]. However, as far as the author knows, the antioxidant properties of these types of carbon dots have not been reported.

*Momordica charantia* (commonly known as bitter melon or bitter gourd) is a medicinal plant widely distributed in tropical and subtropical regions and extensively used in traditional medicine across sub-Saharan Africa and Asia. It has been employed for the treatment of diabetes, gastrointestinal disorders, malaria, viral infections, and inflammatory conditions, many of which are linked to oxidative stress [20]. Phytochemical investigations have revealed that *M. charantia* contains a diverse array of bioactive compounds, including cucurbitane-type triterpenoids (e.g., momordicosides and charantins), flavonoids, phenolic acids, alkaloids, and polypeptide-p (a plant-derived insulin-like peptide) [21,22]. Recent studies further suggest that extracts and fractions from *M. charantia* modulate redox signalling pathways and enhance endogenous antioxidant defences, reinforcing its role as a promising natural therapeutic against oxidative stress-related diseases [23]. Given its dual status as both a food and medicinal plant, *M. charantia* provides a strong rationale for its investigation as a source of safe, plant-derived antioxidants in this study.

## 2. Materials and Methods

### 2.1. Materials

Citric acid, ethylenediamine, formamide, and iron(iii)chloride hexahydrate were purchased from Sigma-Aldrich (Johannesburg, South Africa). Whereas acetone and ethanol were purchased from Enterprise Solvents (Pretoria, South Africa). *Momordica charantia* leaves were sourced from Nigeria. Trichloroacetic acid, 2,2-diphenyl-1-picrylhydrazyl hydrate, ferric chloride and ascorbic acid were purchased from Sigma-Aldrich. Methanol, potassium ferricyanide, disodium phosphate, dihydrogen phosphate, and ammonium molybdate were of analytical grade and purchased from reputable commercial suppliers.

### 2.2. Methods

#### 2.2.1. Synthesis of Carbon Dots (CDs)

##### Synthesis of Carbon Dots Derived from Citric Acid Precursor (CB-Ca)

The synthesis was carried out following our previous synthesis with slight modification [24]. Briefly, 1.2 g of citric acid (CA) and 2 mL of ethylenediamine were dissolved in 50 mL of formamide to form a transparent solution. The solution was then transferred into a Teflon-lined stainless steel autoclave, placed in an oven, and heated at 180 °C for 4 h. After the synthesis, the autoclave was cooled to room temperature. The dark red solution obtained was filtered with filter paper and 0.22 micron syringe filters to remove larger particles. Then, acetone was added to precipitate the formed carbon dots, which were centrifuged at 9500 rpm for 10 min and washed twice with a mixture of acetone and ethanol (1:1). The obtained CB-Ca was dried under a fume hood.

##### Synthesis of Iron-Doped Carbon Dots (CB-Fe)

The above synthesis was repeated, whereby 1.2 g CA and 2 mL ethylenediamine were dissolved in 50 mL formamide. Then 116 mg iron (iii)chloride hexahydrate was added, and the solution was transferred into a Teflon-lined stainless steel autoclave and heated at 180 °C for 4 h in an oven. The purification procedure above was repeated.

##### Synthesis of Carbon Dots Derived from *Momordica charantia* Leaves (CB-Mc)

About 3 g *Momordica charantia* leaves were soaked in 40 mL ethanol for 4 h in a closed beaker under slow magnetic stirring. The ethanolic extracts were filtered using a filter paper, transferred into a beaker, put in a microwave and irradiated at full power (700 W) for 15 min. The brownish solid formed was cooled to room temperature, and ethanol was added to the beaker to dissolve the synthesised CB-Mc.

#### 2.2.2. Characterisation of CDs

(a) Absorption characterisation using Ultraviolet-visible (UV-Vis) spectroscopy

The UV-vis absorption data were obtained using a Perkin Elmer (Waltham, MA, USA), Lambda 25 spectrophotometer with the absorption wavelength range of 200 to 700 nm. For this characterisation, a small mass of each sample was dissolved in deionised water, diluted to 3 mL and transferred into the UV-vis cuvette cell and put in the instrument. The deionised water was used as a blank or reference for all the absorption characterisations. After the first analysis, the material was further diluted until reaching the absorbance of 0.1 at the maximum absorption position to obtain a smooth curve and for other optical characterisations such as photoluminescence.

(b) Photoluminescence (PL) spectroscopy

The PL analyses were carried out using Shimadzu RF 6000 spectrophotometer with a scan range of 200–900 nm. The standardised sample solutions with the absorbance of 0.1 at the maximum absorption wavelength were taken immediately after UV-vis characterisation into the PL cuvette and excited at different wavelengths from the UV to NIR range to obtain the maximum emission position and other fluorescence properties.

(c) Fourier Transform Infrared (FTIR) spectroscopy

The surface chemistry of the as-synthesised materials was investigated using FTIR spectroscopy. A PerkinElmer Spectrum Two Universal Attenuated Total Reflection (UATR) spectrometer was used for the identification of the functional groups. A small amount of each dried sample was placed on the instrument and pressed until the noise was reduced, and scanned from 400 to 4000 cm^−1^.

(d) X-ray Powder Diffraction (XRD)

The XRD patterns of the as-synthesised materials were obtained by putting an adequate amount of the dried sample on the sample holder and placing it in a Bruker D8 Focus diffractometer with a scanning range of 2ϴ from 0° to 90°. The data were saved and plotted using Origin 8.5 software.

(e) High Resolution Transmission Electron Microscopy (HRTEM)

The morphological analyses were carried out using JEOL 2010 HRTEM operating at 200 kV. Briefly, an adequate amount of each sample was dissolved in deionised water to form a dilute solution. Then, a drop was deposited onto the carbon-coated copper grid and dried overnight for anaylsis.

#### 2.2.3. Antioxidant Assays

(a) 2,2-diphenyl-1-picrylhydrazyl (DPPH) radical scavenging activity

The DPPH assay was carried out following the method originally outlined by [25], as modified by [26]. To perform the assay, stock solutions of the quantum dots were prepared and then diluted to reach the desired final concentrations using 98% methanol as the diluent. As a standard reference, ascorbic acid was used. In this assay, the bleaching rate of DPPH was monitored at a wavelength of 517 nm using a UV-Vis spectrophotometer. The test samples, in varying volumes, were adjusted to a final volume of 0.5 mL with 98% methanol. Subsequently, 0.5 mL of a 0.3 mM DPPH solution in 98% methanol was introduced into the test tube. The resulting mixture was thoroughly mixed and incubated in the dark at room temperature for 20 min. Following this incubation period, the absorbance of the solution was read at 517 nm against a DPPH control solution, which contained 0.5 mL of methanol in place of the test samples.

The percentage of scavenging activity was calculated using the following formula [26]:

$$Scavenging\;activity\;\%=\frac{{Abs}_{\left(Control\right)}-{Abs}_{\left(Sample\right)}}{{Abs}_{\left(Control\right)}}\times 100$$

where

Abs_(control)_ is the absorbance of the control (containing all reagents except the test sample) and

Abs_(sample)_ is the absorbance of the test compound/standard.

Data obtained were analysed using non-linear regression curve for the determination of the IC_50_ on GraphPad Prism 9.4 (GraphPad Software Inc., San Diego, CA, USA).

(b) Hydrogen Peroxide Scavenging Effects

The capacity of the carbon dots to scavenge hydrogen peroxide was determined following the method established by [27]. A solution of hydrogen peroxide at a concentration of 1.77 M was prepared in 50 mM phosphate buffer pH 7.4. Various concentrations of the quantum dots were prepared in 0.5 mL of the hydrogen peroxide solution. The absorbance of this reaction mixture was recorded at 230 nm in a UV spectrophotometer. A solution of hydrogen peroxide prepared in the buffer, without the nanomaterial, served as the control. The degree of hydrogen peroxide scavenging was computed based on the procedure outlined by [28].

$$Scavenging\;activity\;\%=\frac{{Abs}_{\left(Control\right)}-{Abs}_{\left(Sample\right)}}{{Abs}_{\left(Control\right)}}\times 100$$

The IC_50_ value was determined as described earlier in the DPPH assay.

(c) Ferric-Reducing Capability Assay

The evaluation of the extent of inhibition of the ferric-reducing ability of the nanomaterials was conducted following the procedure outlined by [29], using ascorbic acid as the reference antioxidant. Various concentrations of quantum dots were mixed with 1 mL of 100 mM sodium phosphate buffer (pH 6.5), followed by the addition of 1 mL of 1% potassium ferricyanide. The reaction mixture was incubated at 50 °C for a duration of 20 min. Subsequently, 1 mL of 10% trichloroacetic acid (*w*/*v*) was added. To separate the precipitate, the mixture was subjected to centrifugation at 3500 rpm for 10 min, following which 0.5 mL of the supernatant layer was mixed with 0.5 mL of distilled water and 0.1 mL of 0.1% ferric chloride. The absorbance of the solution was read at 700 nm. The reducing power was quantified in terms of equivalent concentration (EC), which is defined as the concentration of the antioxidant that provides a ferric-reducing ability equivalent to that of the reference.

(d) Total Antioxidant Capacity

The total antioxidant capacity of the nanomaterials was measured by the phosphomolybdenum method using ascorbic acid as a standard [30]. A 0.1 mL aliquot of the sample solution was mixed with 1 mL of phosphomolybdate reagent (0.6 M sulphuric acid, 28 mM sodium phosphate and 4 mM ammonium molybdate) while stirring. The test tubes were covered and incubated in a water bath at 95 °C for 90 min. After cooling to room temperature, the absorbance of the mixture was read at 695 nm. Total antioxidant capacity was expressed as mg equivalents of ascorbic acid/g of the sample.

## 3. Results

### 3.1. Optical Characterisation of CB-Ca, CB-Fe and CB-Mc

(a) Absorption analysis

**Figure 1:**
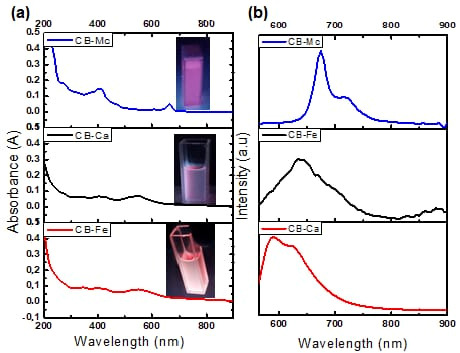
(**a**) Absorption and (**b**) PL spectra of CB-Ca, CB-Fe and CB-Mc.

The absorption and emission properties of the carbon-based nanomaterials were measured using a UV-Vis spectrophotometer and photoluminescence spectroscopy (PL). Figure 1a shows absorption spectra of the as-synthesised materials, and the insets are their respective images under the UV lamp irradiation. The CB-Mc shows absorption peaks at 420 and 650 nm that may be attributed to the π–π* transition of electrons and overlapping weak n–π* absorptions of the carbonyl, –OH, and other functional groups present in the prepared material. The CB-Fe shows prominent peaks at 350, 400 and 550 nm, signifying the presence of highly conjugated double bonds and n–π* indicating the carbonyl and nitrogen-doped carbon structures. In addition, the CB-Ca displays similar peaks as CB-Fe, but with strong absorptions due to the absence of iron on the surface of the quantum dots.

(b) PL emission analysis

Figure 1b shows the PL spectra of the three carbon dots when excited at 550 nm. The CB-Mc shows high emission at 675 and 720 nm. This correlates with the absorption peak at the longer wavelength and is attributed to the presence of many different atoms, such as sulphur and nitrogen, on the surface of the quantum dots from the leaves. On the other hand, CB-Ca shows emission at 590 and 625 nm. However, the addition of iron red shifted the emission to 640 nm. The addition of iron alters the energy states within the band gap and leads to a smaller energy gap as compared to the initial one. This addition quenches the emission and enhances the trap-state, resulting in a red-shifted emission [31].

### 3.2. Structural Characterisation of CB-Ca, CB-Fe and CB-Mc

(a) Fourier Transform Infrared (FTIR) spectra

**Figure 2:**
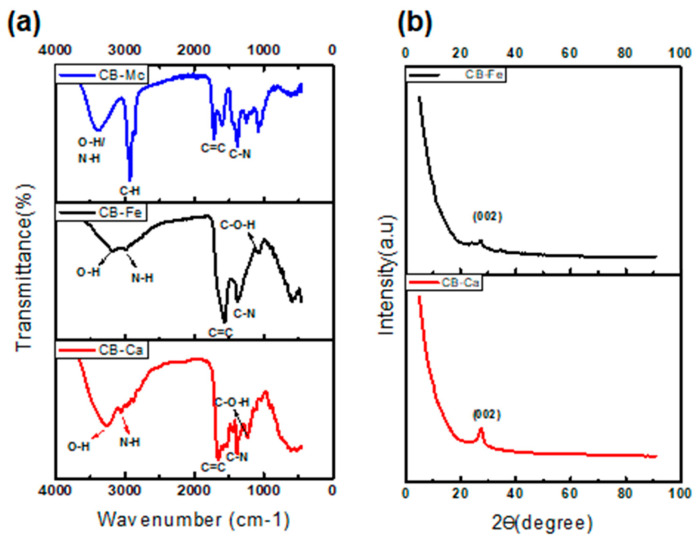
FTIR spectra (**a**) and XRD patterns (**b**) of the as-synthesised carbon dots.

The spectrum for CB-Ca (Figure 2a) shows an O-H band at 3260 cm^−1^ and stretching vibration bands at 2989–2899 cm^−1^, which are attributed to the N-H group of carbonised citric acid. The bands at 1650 and 1384 cm^−1^ are linked to the bending vibration absorption bands of C=C and C-N, respectively.

The CB-Fe spectrum (Figure 2a) shows absorption bands at 3191–2990 cm^−1^ assigned to O-H and N-H. These indicate an abundance of amino and hydroxyl groups on the surface of CBQDs, which resulted in good hydrophilic properties of the CBQDs. The bands at 1571 and 1370 cm^−1^ are attributed to the bending vibration absorption bands of C=C and C-N, respectively. The C-O-H and C-H bands were visible at 1056 and 762 cm^−1^. The presence of hydrophilic functional groups in the CB-Ca and CB-Fe leads to their high solubility.

The FTIR spectrum of CB-Mc (Figure 2a), shows a broad peak at 3394 cm^−1^, confirming the presence of an O-H group, whereas the peak at 2924 cm^−1^ indicates the presence of N-H stretching, and the peak at 1716 cm^−1^ points toward the presence of C=C functionality in the CB.

(b) X-Ray Diffraction (XRD) analysis

Both CB-Ca and CB-Fe display crystalline nature and show a broad peak at 2θ = 27.7 and 26.6 °, respectively, ascribed to Bragg’s reflection of the (002) carbon in the graphene layers that is similar to graphite [32]. This peak usually represents a measure of interplanar spacing for graphene sheets, and it nearly disappears after doping CB with iron. The minor peak at 35 ^o^ in Ca-Fe indicates the presence of iron and is also associated with the traces of iron oxide [31].

(c) Dynamic Light Scattering (DLS)

The average size of the as-synthesised CBs ranges from 3.9–6.0 nm (Table 1). The surface charge of the CB-Ca and CB-Mc is negative due to the abundance of N and S atoms attached to the surface of the carbon dots. However, the doping with iron changed the potential to positive due to the cationic nature of iron (iii).

(d) High Resolution Transmission Electron Microscope (HRTEM)

**Figure 3:**
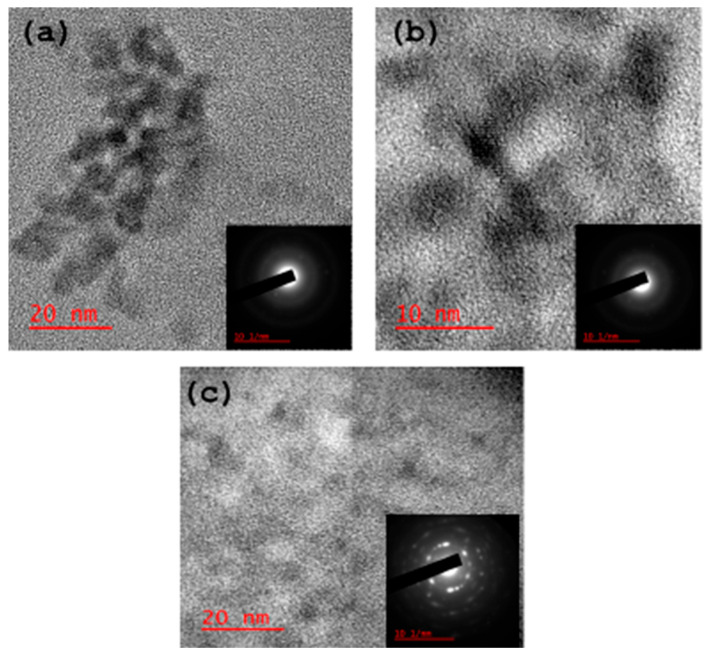
HRTEM micrographs of the carbon dots: (**a**) CB-Ca, (**b**) CB-Fe and (**c**) CB-Mc.

The HRTEM micrographs (Figure 3) show that all the as-synthesised carbon dots are spherical and crystalline. The respective selected area electron diffraction (SAED) also confirmed the crystalline properties of the materials.

### 3.3. DPPH Radical Scavenging Effect

**Figure 4:**
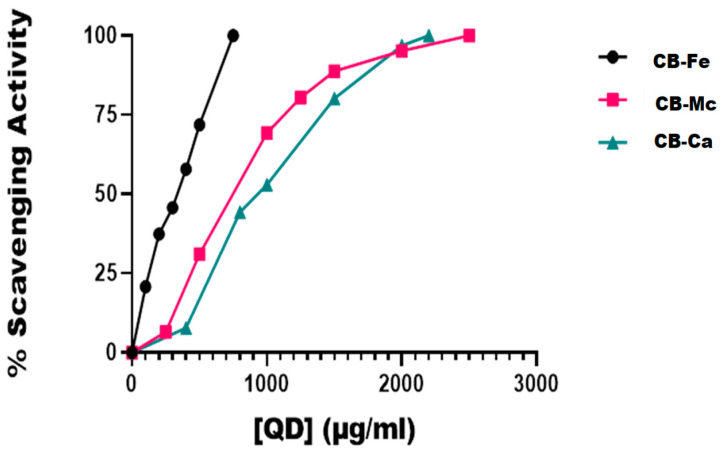
In vitro DPPH radical scavenging activities of the different carbon-based quantum dots. Abbreviations: CB-Fe: iron-doped carbon dots, CB-Mc: carbon dots derived from *Momordica charantia* leaves, and CB-Ca: carbon dots derived from citric acid precursor.

Figure 4 is a plot of the DPPH radical scavenging activity of the three carbon-based nanomaterials. At 100 µg/mL, iron-doped carbon dots (CB-Fe) had the highest radical scavenging activity of about 20%. The other two-carbon dots derived from *M. charantia* leaves (CB-Mc) and carbon dots derived from citric acid precursor (CB-Ca) had almost the same percentage activity (5%). The materials showed a good ability to donate protons for free radical quenching. Overall, CB-Fe exhibited the highest scavenging activity, as evidenced by its IC_50_ value of 254.2 ± 37.37 µg/mL. In contrast, CB-Mc and CB-Ca showed comparatively lower scavenging activities with values of 513.1 and 678.4 µg/mL, respectively. Furthermore, at concentrations of 1000 µg/mL, both CB-Mc and CB-Ca were nearing the maximum limit of their scavenging activity whereas CB-Fe remains unsaturated, suggesting that CB-Fe possesses superior scavenging ability. It was however, observed that the standard, ascorbic acid gave the best scavenging property with IC_50_ value of 1.9 ± 0.26 µg/mL.

### 3.4. Hydrogen Peroxide Scavenging Activity

Table 2 shows the summary of the hydrogen peroxide scavenging ability of the various carbon-based nanomaterials. CB-Mc had the highest value of about 1500 µg/mL, followed by CB-Fe, indicating that the CB-Ca demonstrated the highest efficiency in neutralising hydrogen peroxide.

### 3.5. Ferric-Reducing Power Ability

The ferric-reducing ability of the carbon-based quantum dots, expressed in terms of ascorbic acid equivalent (AAEq) is presented inFigure 5. CB-Fe exhibited the highest antioxidant activity with a value of 58.22 ± 7.35 mg AAEq/g, while CB-Ca also had significant antioxidant potential with a value of 48.6 ± 0.13 mg AAEq/g. In contrast, CB-Mc showed the least antioxidant ability (23.39 ± 0.95 mg AAEq/g) among the tested materials.

### 3.6. Total Antioxidant Capacity

**Figure 6:**
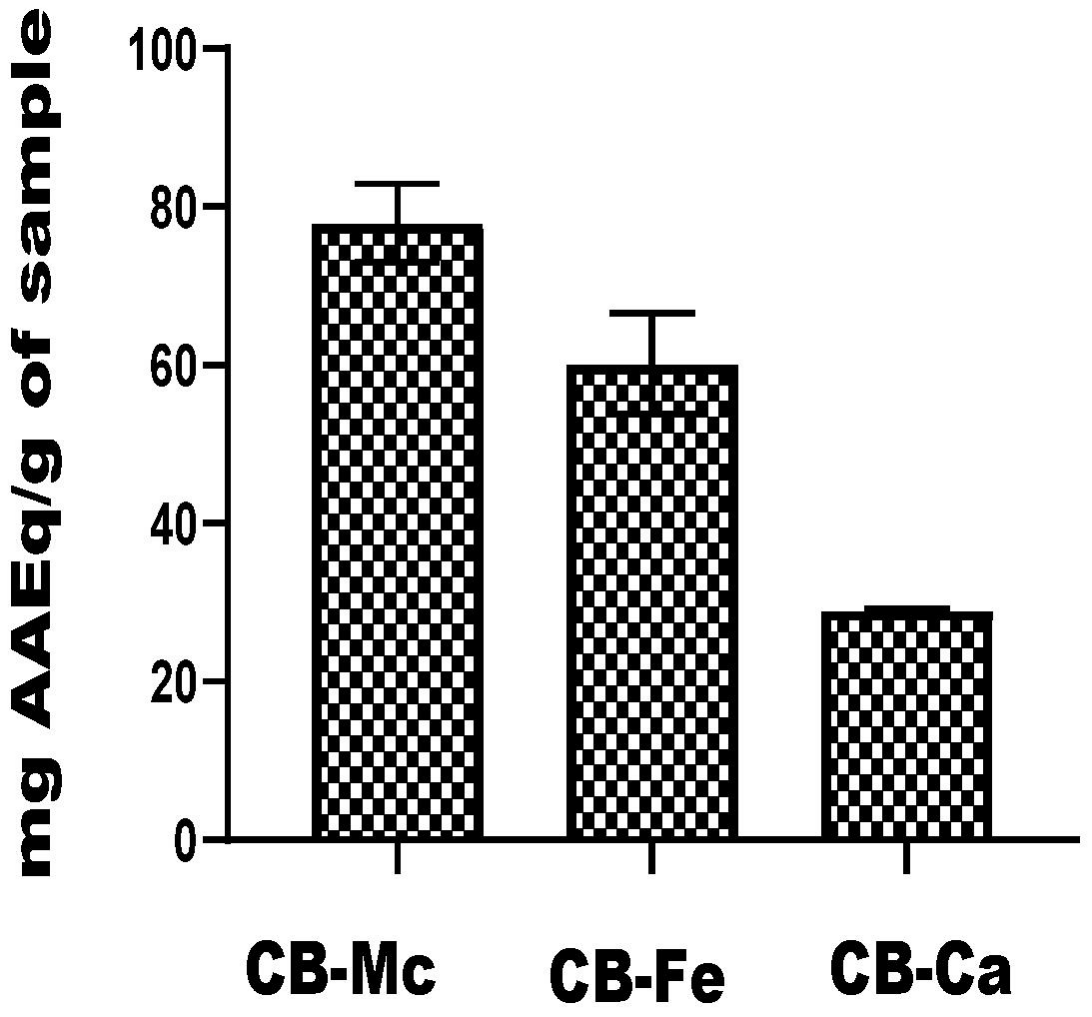
Total antioxidant capacity of carbon-based nanomaterials. Abbreviations: CB-Fe: iron-doped carbon dots, CB-Mc: carbon dots derived from *Momordica charantia* leaves, and CB-Ca: carbon dots derived from citric acid precursor.

The total antioxidant capacity of the carbon-based nanomaterials is shown in Figure 6. The CB-Mc exhibited the highest capacity, with a value of 77.95 mg ± 4.95 AAEq/g, while CB-Fe also showed a substantial antioxidant ability, with a value of 60.19 ± 6.39 mg AAEq/g. It was, however, observed that CB-Ca exhibited the lowest antioxidant potential among the three nanomaterials, with a value of 29.07 ± 0.19 mg AAEq/g.

## 4. Discussion

The antioxidant capabilities of carbon dots derivates; undoped carbon dots and iron-doped carbon dots using citric acid as a precursor and novel carbon dots derived from *Momordica charantia* leaves, are reported in this work in anticipation of their possible application in the management of stress-related diseases associated with reactive oxygen and nitrogen radicals.

Among the materials tested, iron-doped carbon dots (CB-Fe) showed the highest capacity to scavenge DPPH radicals, with an IC_50_ value of 254.2 ± 37.37 µg/mL (Figure 4). Given its ability to neutralise free radicals, this suggests that CB-Fe could be useful in applications requiring antioxidant properties. CB-Fe has a good capacity to scavenge DPPH, as it was not easily saturated with reactive radicals even at high concentrations compared to other nanomaterials (CB-Mc and CB-Ca) that were saturated with reactive radicals as the nanomaterials’ concentrations approached 1000 µg/mL.

On the other hand, the undoped carbon dots (CB-Ca) displayed the highest hydrogen peroxide scavenging activity with an IC_50_ value of 84.2 ± 11.87 µg/mL. This suggests that CB-Ca could be effective at mitigating the damaging effects of hydrogen peroxide that are often generated during metabolic reactions, particularly in the electron transport chain. It is noteworthy that a far higher concentration of CB-Mc is needed to achieve the same effect as CB-Ca. These findings highlight how various carbon-based materials could have differing specificities in their response to various forms of reactive radicals generated through different mechanisms.

In the assessment of total antioxidant capacity, CB-Mc with a value of 77.95 ± 4.95 mg AAEq/g, showed the highest total antioxidant capacity. This finding implies that carbon dots derived from *M. charantia* probably possess the most potent antioxidant properties out of all the materials examined. Conversely, CB-Ca showed the lowest overall antioxidant capacity. An evaluation of the materials’ capacity for electron transfer is given by their ability to reduce ferric tripyridyltriazine to a ferrous form. CB-Fe demonstrated the best ability in this particular analysis, demonstrating its superior redox and electron transfer capabilities. These results further emphasise how different carbon-based materials have different antioxidant potentials, highlighting how important it is to choose appropriate nanomaterials for a given application.

Although ascorbic acid and Trolox remain standard references for antioxidant potency, their utility is constrained by inherent limitations. Both are consumed stoichiometrically during radical quenching and are prone to oxidative degradation that reduces stability during storage and delivery [33]. Vitamin C, despite its strong radical-scavenging efficiency (IC_50_ = 1.9 ± 0.26 µg/mL in DPPH and IC_50_ = 24.2 ± 7 µg/mL in hydrogen peroxide assays), rarely exceeds plasma concentrations of ~80 µM even under high supplementation [34]. This discrepancy between in vitro potency and in vivo availability represents a fundamental limitation, as physiological levels cannot replicate the radical-quenching capacity observed under assay conditions. Moreover, these small molecules act largely through single-mode scavenging, whereas carbon-based nanomaterials exhibit tuneable redox behaviour, sustained or enzyme-mimetic activity, and multifunctionality [35,36]. Such features suggest that nanomaterials provide mechanistic advantages that conventional antioxidants cannot achieve.

In addition, the ability to generate carbon dots from inexpensive and locally available biomass such as *M. charantia* leaves, combined with the versatility offered by metal doping, positions these materials as scalable candidates for further development. While their present potency remains lower than classical standards, the modularity, stability, and multifunctionality of carbon-based nanomaterials argue strongly for their continued optimisation as next-generation antioxidants.

## 5. Conclusions

In this study, we reported the synthesis of three nontoxic carbon-based nanomaterials—carbon dots from citric acid precursor, iron-doped carbon dots and carbon dots derived from *Momordica charantia* leaves—and examined their antioxidant capabilities using different antioxidant assays, including 2,2-diphenyl-1-picrylhydrazyl (DPPH) radical scavenging, hydrogen peroxide (H_2_O_2_) scavenging, ferric-reducing antioxidant power, and total antioxidant capacity (TAC) assays. The optical and structural analyses confirmed the synthesis, morphology and crystalline nature of the as-synthesised carbon-based nanomaterials. The antioxidant assays show the significance of choosing the right carbon-based material in accordance with the intended antioxidant or redox-related application. Iron-doped carbon dots showed excellent electron transfer capabilities and remarkable DPPH scavenging activity. Carbon dots derived from *M. charantia* had the highest total antioxidant capacity, whereas carbon dots derived from citric acid were highly effective in neutralising hydrogen peroxide.

## Figures and Tables

**Figure 5:**
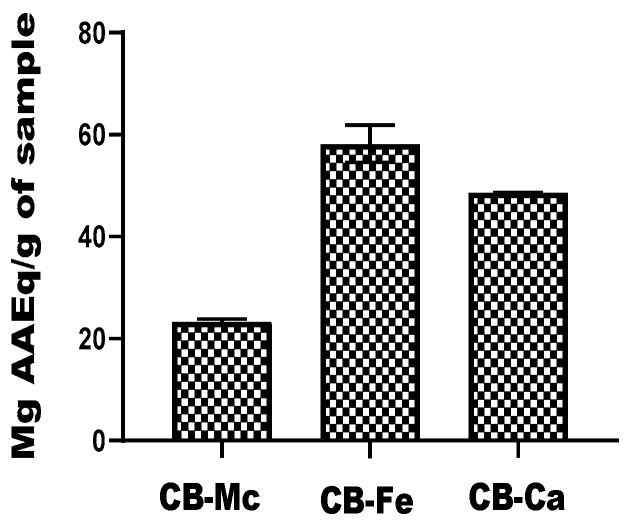
Comparison of reducing power efficiency of carbon-based nanomaterials. Abbreviations: CB-Fe: iron-doped carbon dots, CB-Mc: carbon dots derived from *Momordica charantia* leaves, and CB-Ca: carbon dot derived from citric acid precursor.

**Table 1:** DLS results of the carbon dots.

Quantum Dots	Size (nm)	Zeta Potential (mV)
CB-Ca	3.98 ± 0.047	−27
CB-Fe	4.60 ± 0.053	+31
CB-Mc	5.99 ± 0.084	−24

**Table 2:** Hydrogen peroxide in vitro scavenging activity of carbon nanomaterials.

Quantum Dots	H_2_O_2_ IC_50_ (µg/mL)
CB-Fe	251.3 ± 22.82
CB-Mc	1501 ± 149.7
CB-Ca	84.2 ± 11.87
Ascorbic acid	24.2 ± 7

The results are shown as the average of three determinations ± SEM.

## Data Availability

Data is contained within the article.
